# A Study on the Cognition and Emotion Identification of Participative Budgeting Based on Artificial Intelligence

**DOI:** 10.3389/fpsyg.2022.830342

**Published:** 2022-03-08

**Authors:** Yuan Zhou, Tianjiao Zhang, Lan Zhang, Zhaoxin Xue, Mingxu Bao, Lingbing Liu

**Affiliations:** ^1^College of Accounting, Jilin University of Finance and Economics, Changchun, China; ^2^School of Innovation and Entrepreneurship, Changchun University of Chinese Medicine, Changchun, China; ^3^School of Accounting, Dongbei University of Finance and Economics, Dalian, China

**Keywords:** cognition, emotion, artificial intelligence, participative budgeting, performance

## Abstract

Cognition and emotion exert a powerful influence on human behavior. Based on cognitive psychology and organizational behavior theory, this paper examines the role of cognition and emotion in participative budgeting and corporate performance using a questionnaire survey. The questionnaires were sent to 345 listed companies in China. The results support the hypothesis that human cognition and emotion have a positive moderating effect on the relationship between participative budgeting and corporate performance. Cognition and emotion can promote the effect of participative budgeting on corporate performance. Furthermore, according to the theory of artificial intelligence (AI), this paper designs an AI-based cognition and emotion identification system. This system can help managers identify the budget participants’ cognitive and emotional states and undertake the interventions necessary to improving corporate performance.

## Introduction

In the postepidemic era, enterprise risk management has become key to achieving sustainable development (Yu et al., 2020). Budgeting is an important tool for enterprise risk control. Participative budgeting prefers that employees at all levels of the organization participate in the preparation and evaluation of the budgeting. As a multiparty budgeting system, the results are often multifaceted. Most studies on the relationship between participative budgeting and corporate performance focus on economics and management, and many scholars believe that participative budgeting can promote corporate performance (Peter and Morris, 1986). However, some scholars put forward a different view, that participative budgeting is negatively correlated or not significantly correlated with corporate performance (Cherrington and Cherrington, 1973). This indicates that there may be a complex interactive mechanism between participative budgeting and corporate performance.

Cognition and emotion may play an important role in the relationship between participative budgeting and corporate performance. As a management tool, participative budgeting is closely related to the participants (Milani, 1975), and the participants’ psychological characteristics affect the result of participative budgeting (Shields and Shields, 1998). Cognition and emotion are important psychological links between organizations and employees. People with high cognitive and emotional abilities are more likely to take positive actions. Employee cognition can affect the identification and transmission of information in budget participation and then influence the quality of decision-making. Employees with great emotional ability usually participate actively in the formulation and implementation of budgeting. Therefore, cognition and emotion may play an important role in the relationship between participative budgeting and corporate performance. Exploring how of cognition and emotion affect participative budgeting and corporate performance will play a positive role in improving corporate performance.

With the development of science, it has become possible to identify human cognition and emotion through artificial intelligence (AI). Connectionism holds that cognition arises from the human nervous system so cognitive processes can be simulated by using mathematical models of the nervous system. At present, researchers have constructed a variety of cognitively and emotionally intelligent identification models, such as the CogAff model (Solman, 2004), EI model (Ryan et al., 2018), MEM model (Yoichiro et al., 2020) among others. These models can imitate cognition and emotion to some extent, and provide a reference for research on the identification of cognition and emotion. Therefore, this paper attempts to construct an AI-based cognition and emotion identification system. Meanwhile, the cognition and emotion of participants in budgeting can be reasonably interfered with through this system to promote corporate performance.

In summary, our study has two contributions. First, we divide cognition and emotion into three important parts, cognitive ability, emotional intelligence and self-efficacy, and verify their positive moderating effects on corporate performance in participative budgeting by empirical analysis. Our results can extend the research scope of cognitive psychology and organizational behavior. Second, an AI-based cognition and emotion identification system is constructed to identify the cognitive and emotional processes of participants in budgeting. Corresponding management suggestions are put forward by this system. Our aim is to help managers identify their employees’ cognitive and emotional problems and implement more valid solutions. The main characteristics of the system are as follows:

(1) A questionnaire data processing system (QDP) is used for matching questionnaire data and cognitive and emotional labels to identify the cognitive and emotional processes of participants in budgeting.
(2) An artificial neural network (ANN) is used for extracting the features of cognition and emotion of participants in budgeting, and a production expert system (PES) is used for identifying cognitive and emotional problems and providing solutions.
(3) The management application innovation system (MAI) combines the solutions provided by machine analysis with the manager’s own wisdom.

## Theoretical Analysis of the Role of Cognition and Emotion in Organizational Behavior and Performance

### Cognition, Emotion and Organizational Behavior

In the continuous development of corporate management practice, managers have been aware that it is impossible to arouse the enthusiasm of workers simply by adopting classical management theory. Exploring the emotional basis in management, that is, seeking the relationship between emotion and behavior in organizations, should be of great importance.

The change in behavior is a process that includes three stages: “knowing” (cognition), “feeling” (emotion) and “action” (behavior) (Westbrook and Oliver, 1991). Cognition is the process of acquiring knowledge and information processing, which is the most basic psychological process of human beings. The human brain accepts the information input from the outside world. Then, the information is processed by the brain and converted into mental activities, thus dominating human behavior. It is the process of information processing, also known as cognitive process. Emotion is a complex and stable physiological evaluation and experience of attitude dominated by cognition. When individuals’ desires and needs are met, it causes positive emotions and thus brings changes to their behaviors. The process of emotion formation occurs through the processes of cognition, restraint and motivation. Restraint is the expression of emotional intelligence. Emotion can motivate individuals by guiding and promoting them to achieve predetermined goals, and the strength of this effect is closely related to self-efficacy. A survey of 77 senior managers found that the more intense the competition in the market is, the more positively correlated the degree of budget participation with management performance and job satisfaction (Vincent et al., 2005). Budget participation enhances communication among group members. Participation enables members to fully understand and share the goals and values of the organization, arouses the sense of responsibility of employees, enhances the cohesion of the team, and thus enhances their commitment to the organization (DeCotiis and Summers, 1987). Therefore, it can be inferred that cognition and emotion can affect individual behavior in the organization.

### Organizational Behavior and Organizational Performance

An organization is a systematic arrangement of people to accomplish a specific mission. With increasingly advanced technical means of interpersonal communication, the spatial barriers between people are getting smaller, which significantly improves the effectiveness of contact between people. Along with the increasing socialization of services, the form and degree of interactions between individuals and organizations are complicated and diversified (Zhang, 2011). Modern management has developed from “material-oriented” management to a “people-oriented” one, from a “discipline” study to a “human behavior” one and from “supervision” management to “incentive” management.

Based on modern organizational behavior theory, participative budgeting is a decentralized and bidirectional management mode in which all the staff actively participate in the company budget. By participating in the budgeting process, employees can integrate their own goals into organizational goals. This fully stimulates the creativity and work enthusiasm of employees and enhances their sense of mission within the organization to improve corporate performance. However, in practice, the effect of participative budgeting on corporate performance is complicated. Human behavior is the manifestation of human psychology, which is dominated by cognition and emotion. People with different cognitive and emotional states tend to behave differently within organizations. Correspondingly, behavioral consequences—the impact on corporate performance— varies. Cognitive and emotional factors are not only the results of the influence of organization on individuals but also the internal source of the effect of individual behavior on organizational performance. Therefore, to influence the behavior of employees, the cognitive and emotional factors of participants need to be considered. Sensible psychological intervention can contribute to taking full advantage of the positive role of participative budgeting.

## Empirical Analysis and Test Results of Cognitive and Emotional Influences on Participative Budgeting

### Research Hypotheses

#### Participative Budgeting and Corporate Performance

The discussion on participative budgeting can be traced back to the 1950s (Argyris, 1952). Most scholars have focused on the relationship between participative budgeting and corporate performance. The study found that employees could have more choices in participative budgeting and feel that they can create a working atmosphere of joint efforts. According to self-determination theory, this voluntary participation can motivate employees to contribute to the budget. In this process, employees become more willing to think deeply and formulate strategic budget goals that are conducive to the long-term development of the organization. As a result, it is likely to reinforce job satisfaction and enhance corporate performance (Chenhall, 1986; Wu et al., 2019; Li et al., 2021). Some scholars believe that the effects of participative budgeting on corporate performance are probably influenced by mediating variables or moderating variables (Li et al., 2021). A study of manufacturing in Hong Kong found that budget participation is positively correlated with management performance under a high level of decentralization (Gul et al., 1995), and this positive correlation is also moderated by budget incentives (Wu et al., 2011). As employees participate in budgets, the restraining effect of inconsistencies in budget participation on performance becomes weaker; that is, corporate performance improves with the decrease in budget participation inconsistency (Clinton, 1999). Other studies have found that involving employees in budgeting can alleviate organizational dysfunction caused by budget compulsion and thus indirectly improve corporate performance (Li and Miao, 2013). Thus, building on the above arguments, we propose that:

H1: Participative budgeting has a significant positive effect on corporate performance.

#### The Moderating Effect of Cognition and Emotion

Processes of cognition and emotion include cognition, constraint and motivation. People’s different emotional capacities may play different roles in the influence of participative budgeting and corporate performance. Therefore, this paper divides cognition and emotion into three aspects, namely, cognitive ability, emotional intelligence and self-efficacy.

As a psychological factor with individual differences, cognitive ability is the ability to process, store and extract information. Employee cognitive ability refers to the ability of employees to accurately identify useful information at work and apply it to decision-making. In people-oriented management, the level of cognitive ability could exert a far-reaching impact on the perception of others’ needs, the maintenance of interpersonal relationships and even the enhancement of team cohesion. Rich cognitive perspectives and information sources can significantly improve employees’ ability to creatively propose solutions to tackle problems. Cognition consists of a variety of elements, such as thought, need, attitude and belief. When these elements conflict, cognitive dissonance will occur. Cognitive dissonance makes employees feel strong work pressure, resulting in low work efficiency and a high turnover rate (Festinger, 1957). The study found that the participation of team members in the discussion is conducive to enhancing the overall complexity of cognition. This enables them to think more deeply from multiple perspectives before making final decisions, thereby improving cognitive consistency and decision-making efficiency (Gruenfeld and Hollingshead, 1993; Liu et al., 2020). In general, people with higher cognitive abilities more precisely perceive changes in interpersonal relationships, which is beneficial for further coordination with others. Creating a good interpersonal atmosphere in budget participation is conducive to realizing team cooperation and improving corporate performance (Che and Qin, 2009). Employees with great cognitive ability can better grasp the nature of the budget goals. They can combine the current situation and their own capabilities to make the budget goals more objective and feasible (Li et al., 2020). The participation of employees with great cognitive ability in budgeting can promote a team’s cognitive awareness of the organization’s goals. In this way, the efficiency of resource allocation and budget transparency can be improved, which reduces unnecessary conflicts (Yang and He, 2020). According to these arguments, we propose the following:

H2a: Cognitive ability positively moderates participative budgeting and corporate performance.

Another important process in cognition and emotion is emotional restraint. Emotional self-discipline refers to the effective control of emotions, which is shown as emotional intelligence. Research has shown that emotional intelligence, based on individuals or the entire team, plays an important role in achieving strategic goals (Zhang, 2012). Moderate emotional excitement can put people in an optimal state, both physically and mentally, and its physiological activation function can lead people to achieve the highest level of performance (Hebb, 1955). As an important psychological feature, emotional intelligence helps individuals maintain good emotional states owing to its effect on emotional regulation. Emotional intelligence is key for individuals to fully utilize their abilities. Emotion can induce behavior through the arousal function. A moderate level of arousal can motivate individuals to achieve set goals and improve their work performance (Yerkes and Dodson, 1908). The level of employees’ emotional intelligence determines their work performance to a certain extent. Employees with high emotional intelligence are capable of accurately detecting, integrating and evaluating information, so they can deeply perceive the budget information required by managers. This makes it easy for them to deliver the results the organization expects (Wong and Law, 2002; Wei et al., 2015). It is found that employees with low emotional intelligence have poorer information absorption and summary abilities. As a result, they have difficulties in effectively exchanging information in the budgetary participation process, which hinders the implementation effect of participative budgeting. Emotional intelligence involves the ability of emotions to guide individuals to achieve predetermined goals. Employees with high emotional intelligence can mobilize and direct their emotions to make them subordinate to a given goal. Therefore, they are not easily affected by negative emotions but actively look for ways to solve problems. In this way, they can better complete the budget targets and promote corporate performance (Goleman et al., 2002). Emotional intelligence develops gradually with the growth of people and can be improved through training and intervention (Bar-On and Parker, 2000). Employees use emotional intelligence, the bond between the emotional system and cognitive system, to manage their emotions, which could exert a certain influence on behavior in the workplace (Liu, 2018). Thus, we propose the following:

H2b: Emotional intelligence positively moderates participative budgeting and corporate performance.

With an important role moderating the effect of cognition and emotion on behavior, emotion-based motivation affects adherence to one’s ideal goals and helps overcome negative emotions. This is called self-efficacy. Self-efficacy is “an individual’s definite belief in his or her ability to successfully accomplish a specific task in a specific situation.” It can mobilize motivation, cognitive resources and a set of actions (Fred, 2003). The study found that employees with high self-efficacy are more actively involved in their work compared to employees with low self-efficacy. They tend to set challenging goals and maintain high levels of commitment while persevering despite difficulties, thus showing better performance (Bandura, 1986; Zhao et al., 2018). A high level of self-efficacy means that employees are confident in their ability to overcome difficulties and have a strong willingness to participate in new things (Lu et al., 2001). Based on this, high self-efficacy employees demonstrate high loyalty and are willing to share new knowledge and experience. When employees with a strong willingness to share and high working ability participate in budget preparation, the exchange, sharing and integration of budget information will be more effective. This makes budget planning more objective and feasible and thus promotes corporate performance. At the same time, some studies have shown that employees with high self-efficacy are often less susceptible to external influences and are more willing to set challenging goals and later put them into practice. They are usually able to withstand challenging workloads and better adapt to organizational innovations, thus better achieving budget goals (Seijts and Latham, 2011; Li and Guo, 2021). Employees with high self-efficacy tend to be passionate about their jobs, which strengthens goal commitments and eventually corporate performance. Accordingly, we propose the following:

H2c: Self-efficacy positively moderates participative budgeting and corporate performance.

### Measurement of Variables

There are five latent variables in this paper, namely, participative budgeting, cognitive ability, emotional intelligence, self-efficacy and corporate performance. Maturity scales are used.

1. Participative budgeting (PB). Participative budgeting was measured by a six-item scale adapted from Maiga (2005), as shown in Table 1.
2. Cognition and emotion mechanism. The cognition and emotion mechanism consists of cognitive ability, emotional intelligence and self-efficacy.

**TABLE 1:** Item of participative budgeting.

Variable	Items
PB (Maiga, 2005)	I am involved in setting all of my budget.
	My superior clearly explains budget revisions.
	I have frequent budget-relate discussions with my superior.
	I have a great deal of influence on my final budget.
	My contribution to the budget is very important.
	My superior initiates frequent budget discussion when the budget is being prepared.

(1) Cognitive ability (CA)

According to Spreitzer, cognitive ability was divided into three dimensions with nine items (Spreitzer, 1995), as shown in Table 2.

**TABLE 2:** Item of cognitive ability.

Variable	Items	
CA (Spreitzer, 1995)	Meaning	The work I do is very important to me.
		My job activities are personally meaningful to me.
		The work I do is meaningful to me.
	Competence	I am confident about my ability to do my job.
		I am self-assured about my capabilities to perform my work activities.
		I have mastered the skills necessary for my job.
	Self-Determination	I have significant autonomy in determining how I do my job.
		I can decide on my own how to go about doing my work.
		I have considerable opportunity for independence and freedom in how I do job.

(2) Emotional Intelligence (EI)

Based on mixed model theory, this paper adopts the scale of Law, K. S, to divide emotional intelligence into four dimensions with 16 items (Law et al., 2004). The specific items are shown in Table 3.

**TABLE 3:** Item of emotional intelligence.

Variable	Items	
EI (Law et al., 2004)	Self-Emotions Appraisal (SEA)	I have a good sense of why I have certain feelings most of the time.
		I have good understanding of my own emotions.
		I really understand what I feel.
		I always know whether or not I am happy.
	Regulation of Emotion (ROE)	I am able to control my temper so that I can handle difficulties rationally.
		I am quite capable of controlling my own emotions.
		I can always calm down quickly when I am very angry.
		I have good control of my own emotions.
	Use of Emotion (UOE)	I always set goals for myself and then try my best to achieve them.
		I always tell myself I am a competent person.
		I am a self-motivating person.
		I would always encourage myself to try my best.
	Others-Emotions Appraisal (OEA)	I always know my friends’ emotions from their behavior.
		I am a good observer of others’ emotions.
		I am sensitive to the feelings and emotions of others.
		I have good understanding of the emotions of people around me.

(3) Self-efficacy (SE)

According to the definition of self-efficacy (Yao, 2008), this paper adopts the scale of Yao, K, which contains 11 items in three dimensions. The specific items are shown in Table 4.

**TABLE 4:** Item of self-efficacy.

Variable	Items	
SE (Yao, 2008)	Work Cognition and Motivation Efficacy	I feel that I was right to be in this profession at the beginning.
		Facing new tasks, I am always confident.
		I like to use new methods or techniques to complete work.
	Interpersonal Communication and Recovery Efficacy	Even in a new or unfamiliar working environment, I don’t feel very anxious.
		I can take the initiative to communicate with people around me, whether I am familiar with him or not.
		I am in good health and can reasonably regulate the emotional problems that arise at work.
		Even if I encounter difficulties in interpersonal communication, I have the confidence to solve it through my own efforts.
	Stress and Innovation Ability Efficacy	When faced with a problem, I can usually find a new and creative solution to the problem.
		At work, I can often put forward reasonable and innovative ideas for the project.
		With my wit, I believe I can deal with unexpected situations.
		I can turn innovative ideas into practical applications.

3. Corporate performance (CP). According to Green and Inman (2005), corporate performance is divided into two dimensions, marketing performance and financial performance, with 7 items. The specific items are shown in Table 5.

**TABLE 5:** Item of corporate performance.

Variable	Items	
CP (Green and Inman, 2005)	Financial Performance	Average return on investment over the past 3 years.
		Average profit over the past 3 years.
		Profit growth over the past 3 years.
		Average return on sales over the past 3 years.
	Marketing Performance	Average market share growth over the past 3 years.
		Average sales volume growth over the past 3 years.
		Average sales (in dollars) growth over the past 3 years.

### Model Construction

**FIGURE 1:**
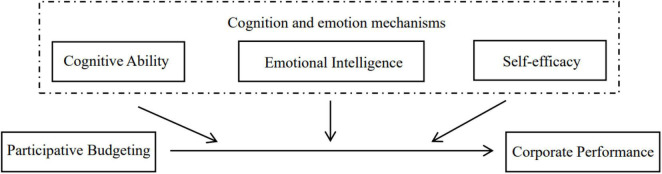
Theory model of cognition and emotion.

Based on the above theoretical analysis and deduction, this paper constructs the following theory model. As shown in Figure 1.

### Questionnaire Design and Data Collection

This paper adopts the questionnaire survey method to collect data, distributed to listed companies in China. We use a five-level scale to score and measure the items. To ensure the accuracy of the questionnaire items, during the formulation of the questionnaire we first invited experts to make preliminary modifications to the questionnaires. Then, we interviewed employees in the enterprise for a second revision. Finally, a small number of MBA students and senior managers of enterprises were tested to form the final draft. A total of 345 questionnaires were sent out, and 320 responses were received. There were 305 valid questionnaires for an effective recovery rate of 88.4%.

### Data Quality Analysis

#### Homologous Deviation Analysis

Harman’s single-factor test was used for homologous bias analysis. Unrelated exploratory factor analysis was performed on the measurement items of all variables by SPSS17.0. The results show that there are 13 factors with eigenvalues greater than 1, and the total variance explained by the first factor was 14.418%, less than the critical value of 40%. Therefore, it can be shown that the homology deviation of the survey data does not affect the final data analysis results.

#### Reliability, Validity and Correlation Test

Table 6 shows descriptive statistics and correlation analysis among variables, namely, the mean value, standard deviation and correlation coefficient. The results showed that participative budgeting (PB), cognitive ability (CA), emotional intelligence (EI), self-efficacy (SE) and corporate performance (CP) were positively correlated. The results provide support for related hypothesis research.

**TABLE 6:** Mean, SD, and correlation coefficient of variables.

Variables	Mean	SD	1	2	3	4	5	6	7	8
1 Corporate scale	3.40	0.84	1							
2 Position of Respondents	4.11	0.84	−0.188**	1						
3 Office term	2.16	1.01	–0.040	–0.086	1					
4 PB	5.08	0.83	–0.062	0.070	–0.001	1				
5 CA	5.11	0.62	–0.030	0.125*	0.039	0.053	1			
6 EI	5.22	0.67	–0.095	0.173**	0.107	0.148**	0.295**	1		
7 SE	5.25	0.59	0.005	0.026	0.017	0.314**	–0.051	–0.003	1	
8 CP	5.36	0.71	−0.192**	–0.011	0.139*	0.255**	0.152**	0.209**	0.254**	1

*p < 0.05 and **p < 0.01.

To test the reliability and validity of all variables, SPSS 22.0 and AMOS 21.0 software were used to test the data. First, the reliability of the data was tested. The results in Table 7 show that the Cronbach’s α value of each variable was greater than 0.7, and the combined reliability CR value was also greater than 0.7, indicating that each variable scale was highly reliable and valid. Second, the validity of the data was tested. In this study, the data were tested by concorporateatory factor analysis (CFA). The results showed that the factor loading value ranged from 0.552 to 0.895, and the average variance extraction value (AVE value) was greater than 0.5, indicating that the scale has good validity. The square root of each variable is greater than the correlation coefficient, indicating that the validity of the scale is high.

**TABLE 7:** Reliability and validity of all variables (*N* = 305).

Variables	Indicators	Factor loading	Cronbach’s α	AVE	CR
PB	PB1	0.749	0.849	0.546	0.878
	PB2	0.774			
	PB3	0.792			
	PB4	0.631			
	PB5	0.751			
	PB6	0.727			
CA	CA1	0.870	0.733	0.678	0.949
	CA2	0.802			
	CA3	0.725			
	CA4	0.737			
	CA5	0.921			
	CA6	0.803			
	CA7	0.755			
	CA8	0.883			
	CA9	0.888			
EI	EI1	0.848	0.852	0.666	0.969
	EI2	0.847			
	EI3	0.862			
	EI4	0.806			
	EI5	0.731			
	EI6	0.875			
	EI7	0.849			
	EI8	0.703			
	EI9	0.796			
	EI10	0.789			
	EI11	0.856			
	EI12	0.780			
	EI13	0.774			
	EI14	0.806			
	EI15	0.843			
	EI16	0.873			
SE	SE1	0.836	0.716	0.689	0.960
	SE2	0.842			
	SE3	0.895			
	SE4	0.856			
	SE5	0.857			
	SE6	0.679			
	SE7	0.726			
	SE8	0.850			
	SE9	0.885			
	SE10	0.858			
	SE11	0.819			
CP	CP1	0.561	0.754	0.549	0.893
	CP2	0.552			
	CP3	0.785			
	CP4	0.738			
	CP5	0.792			
	CP6	0.861			
	CP7	0.834			

### Moderating Effect Test

In this paper, model 1, 2, 3 and 4 were constructed according to the following formulae to test the moderating effect.


(1) $${\text{CP}}_{\text{i}}=\beta_{11}+\beta_{12}\text{Controls}+\varepsilon_{\text{i}}$$

(2) $${\text{CP}}_{\text{i}}=\beta_{21}+\beta_{22}{\text{PD}}_{\text{i}}+\beta_{23}\text{Controls}+\varepsilon_{\text{i}}$$

(3) $${\text{CP}}_{\text{i}}=\beta_{31}+\beta_{32}{\text{PD}}_{\text{i}}+\beta_{33}{\text{CA}}_{\text{i}}+\beta_{34}{\text{EI}}_{\text{i}}+\beta_{35}{\text{SE}}_{\text{i}}+\beta_{36}\text{Controls}+\varepsilon_{\text{i}}$$

(4) $${\text{CP}}_{\text{i}}=\beta_{41}+\beta_{42}{\text{PD}}_{\text{i}}+\beta_{43}{\text{CA}}_{\text{i}}+\beta_{44}{\text{EI}}_{\text{i}}+\beta_{45}{\text{SE}}_{\text{i}}+\beta_{46}{\text{PD}}_{\text{i}}\times {\text{CA}}_{\text{i}}+\beta_{47}{\text{PD}}_{\text{i}}\times {\text{EI}}_{\text{i}}+\beta_{48}{\text{PD}}_{\text{i}}\times {\text{SE}}_{\text{i}}+\beta_{49}\text{Controls}+\varepsilon_{\text{i}}$$

In this study, multiple linear regression was used to test the moderating effects of cognitive ability (CA), emotional intelligence (EI), and self-efficacy (SE) on participative budgeting (PB) and corporate performance (CP) (Models 2, 3, and 4). Model 1 shows the relationships between the control variables and dependent variables. According to Table 8, the effect of participative budgeting on corporate performance (Model 2, β = 0.248, *p* < 0.001) had a significant positive promoting effect. H1 was verified. Model 3 (β = 0.116, β = 0.140, *p* < 0.05 and β = 0.212, *p* < 0.001) and Model 4 (β = 0.122, β = 0.116 and β = 0.120, *p* < 0.05) demonstrated the moderating effects of cognitive ability (CA), emotional intelligence (EI) and self-efficacy (SE), which played a positive moderating role between participative budgeting (PB) and corporate performance (CP) and thus verified H2a, H2b and H2c, as shown in Table 8.

**TABLE 8:** Regression analysis results (*N* = 305).

Variables	CP			
	Model 1	Model 2	Model 3	Model 4
Corporate scale	−0.194**	−0.181**	−0.180**	−0.189***
Position of Respondents	–0.036	–0.051	–0.091	−0.111*
Office term	0.128*	0.128*	0.102	0.088
PB		0.248***	0.157**	0.144*
CA			0.116*	0.109*
EI			0.140*	0.125*
SE			0.212***	0.205***
PB × CA				0.122*
PB × EI				0.116*
PB × SE				0.120*
R2	0.055	0.116	0.190	0.239
Adjusted R2	0.046	0.105	0.171	0.213
F	5.895**	9.872***	9.978***	9.227***

*p < 0.05; **p < 0.01; and ***p < 0.001.

### The Empirical Results

Hypothesis 1, Hypothesis 2a, Hypothesis 2b and Hypothesis 2c in this study are all valid, which proves that cognitive ability, emotional intelligence and self-efficacy have a positive moderating effect on the relationship between participative budgeting and corporate performance. That is, cognitive and emotional factors can promote the positive effect of participative budgeting on corporate performance. It provides a theoretical basis for constructing an artificial intelligence identification system of cognition and emotion.

## Artificial Intelligence Identification System of Cognition and Emotion Based on Participative Budgeting

### Overview of the Development of Artificial Intelligence

In recent decades, artificial intelligence technology has made great progress. Artificial intelligence (AI) began as a simple computer program in the 1950s and gradually evolved to be capable of automatic recovery and self-analysis. By the 1980s, AI gained learning and cognitive abilities (Yao, 2018). Since the beginning of the 21st century, the development of society has shown an accelerating trend of increased connection between sectors due to the widespread use of the internet. The exponential rise of the scale of data creation in the new century has shown a new era of big data thanks to the rapid development of AI. As a tool to explore, develop and extend human intelligence, AI aims to unravel the mystery hidden behind intelligence and develop machines with intelligent response systems similar to human behavior, such as intelligent robots, image recognition technology and more. The emergence and development of AI is changing or even subverting the human way of life and thereby leading society into a new era of connected intelligence.

### Cognition and Emotion Identification Method of Artificial Intelligence

Scholars have carried out many studies about the comprehensive artificial machine simulation of human cognitive and emotional activities over nearly a century, using the knowledge of psychology, neuroscience, neurophysiology, computer science and so on. From the perspective of information science, identifying, understanding and simulating psychological states can be achieved by quantifying psychological information. Furthermore, AI can recognize and process information based on numerical values by constructing a cognitive and emotional information processing model.

With the development of analog neuron technology, artificial neural networks have gradually been used to identify human cognition and emotion. Artificial neural networks (ANNs), information processing systems based on the structure and function of neural networks of the human brain, perform the functions of distributed storage, associative memory and self-learning. Human psychology is a non-linear complex system with uncertainties and non-linearity, while ANNs can activate the processing units to perform non-linear operations. Thus, the study of human psychology by adopting the technology of ANNs is worth exploring. From the perspective of artificial intelligence psychology, it is possible to identify mental activities more accurately by exploring a system (artificial brain) that controls all kinds of behaviors (Newell, 1992). Neural network simulation refers to the design of electronic circuits to simulate neural networks in the human brain. This method can realize part of the brain’s thinking function. By simulating the neuronal path of processing uncertain information, it is found that the decision-making process of humans tends to follow the rule of optimal Bayesian computation, which lays a foundation for the simulation of brain information processing. Some studies build a hierarchical generative model to simulate the neural efficiency of the human cortex. The model can form top-down perceptual data flow and thus simulate the processes that occur when the brain faces uncertainty (Clark, 2013). Some scholars have established an artificial psychological model by applying RBF neural networks, which are composed of three layers of neurons: an input layer, a hidden layer and an output layer. This model can extract a variety of information features and perform non-linear transformation. Then, users’ psychological values can be used to realize the recognition and prediction of their psychological states (Wang, 2017). As a function of the brain, psychology is the reflection of objective reality in the brain. Thus, analog neuron technology is of great significance to psychological identification. At present, some models have imitated mental activities to a limited extent. From the perspective of engineering science, a perfect description and analysis of all mental activities are difficult to realize. Recognizing and simulating limited mental activities are also valuable and meaningful.

### Construction of the Artificial Intelligence-Based Cognition and Emotion Identification System

Through the above analysis, employees’ cognitive ability, emotional intelligence and self-efficacy play an important moderating role in the relationship between participative budgeting and corporate performance. The cognitive and emotional states of employees in these three aspects affect the level of corporate performance. Therefore, it is necessary to build an AI-based cognition and emotion identification system that makes full use of employees’ cognitive and emotional information to realize the automatic identification and intervention of employees’ cognitive and emotional states.

**FIGURE 2:**
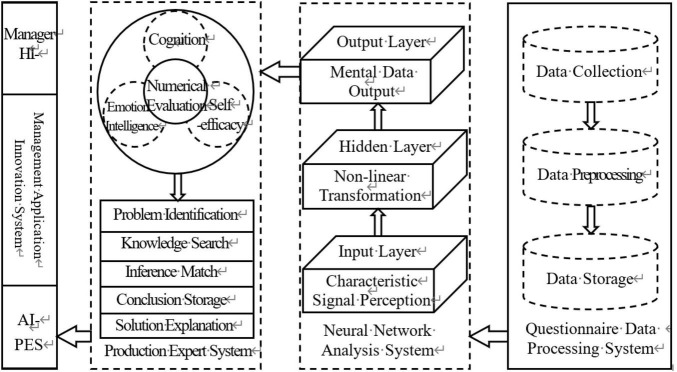
AI-based cognition and emotion identification system.

According to artificial intelligence psychology, psychology is the natural reflection of the brain. AI can recognize the processes of mental activity by simulating the information processing of brain activity. It is known that the perception, analysis, and evaluation of information by the brain relies on neural networks to function. Intelligent models based on the nervous system, such as artificial neural networks, can understand overall brain activity through the connection of each processing unit, thereby mapping the psychological characteristics. In this paper, by combining data processing technology, radial basis function neural network technology and expert system tools, we built an idealized system to simulate the process of cognitive and emotional information acquisition, recognition, analysis, expression and application based on affective computing research. We try to simulate the processes intelligently through four subsystems: a questionnaire data processing system, neural network analysis system, production expert system and management innovation application system. We expect to realize the intelligent identification of employees’ cognitive and emotional states based on the simulation of brain functions and provide some inspiration for management practices. Based on this, we constructed an AI-based cognition and emotion identification system, as shown in Figure 2.

#### Questionnaire Data Processing System

**FIGURE 3:**
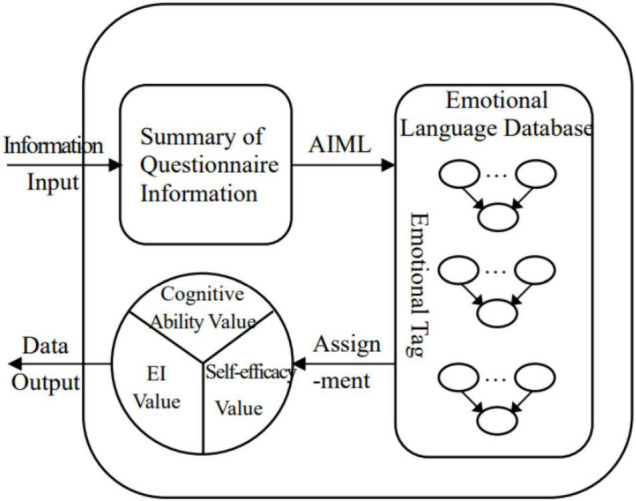
Schematic diagram of data preprocessing module.

The questionnaire data processing system consists of a data collection module, a data preprocessing module and a data storage module. Among them, the data collection module collects and records data about the employees’ cognitive and emotional characteristics by designing questionnaires. As a subjective experience measurement method, the questionnaire can directly obtain the cognitive and emotional states of employees and avoid the measurement errors caused by inferring mental activities through behaviors in intelligent recognition (Picard, 2003). The data preprocessing module matches and classifies the collected cognitive and emotional information. The module is embedded with a perceptual language database, which mainly collects and summarizes adjectives that reflect the cognitive and emotional characteristics of employees in budgeting. Relying on artificial intelligence markup language (AIML), different cognitive and emotional characteristics are designated with emotional tags, such as “confident” to describe self-efficacy. By matching the questionnaire data with emotional tags, the topic information is classified and summarized, and the characteristics of employees’ cognitive ability, emotional intelligence and self-efficacy are formed and assigned. Finally, valid data are input into the data storage module to provide support for later retrieval and analysis, as shown in Figure 3.

#### Neural Network Analysis System

**FIGURE 4:**
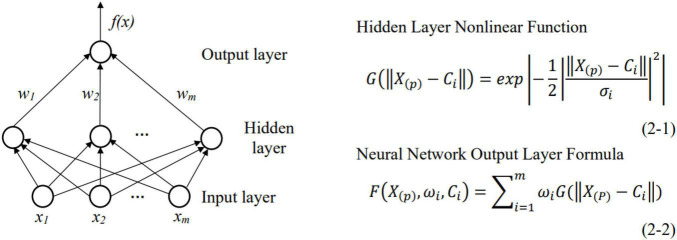
Radial basis function neural network structure diagram.

Adopting ANNs—the adaptive dynamic system composed of non-linear processing units—for information processing and pattern recognition can move technology closer to human thinking. Based on a radial basis function neural network, this system is a feedforward neural network with multiple inputs and a single output, which uses multidimensional data feature values as network inputs and employees’ mental values as outputs. The neural network has a strong non-linear fitting ability and self-learning ability and can better simulate the complex non-linear system of human psychology (Wang, 2017). As a three-layer feedforward neural network, it is composed of an input layer, a hidden layer and an output layer, as shown in Figure 4. The input layer consists of signal source nodes, in which the neurons are responsible for sensing and transmitting the inputs of employees’ cognitive and emotional characteristics. After initial identification of the information, it is transmitted to the hidden layer, which extracts the cognitive and emotional feature values and uses the Gaussian function to perform non-linear conversion, as shown in Formula 2-1. In the formula, *X*_(_*_*p*_*_)_ = (*x*_*p1*_, *x*_*p2*_…, *x*_*pm*_) is the *p-th* input sample in the cognitive and emotional feature sample set, and *C*_*i*_ = (*c*_1_, *c*_2_…, *c*_*n*_) is the center of the *i-th* hidden layer neuron. *σ^2^* is the normalized parameter of the *i-th* hidden layer node. The output layer linearly combines the values of non-linear cognitive and emotional characteristics to output the data on the cognitive and emotional values of employees, such as cognitive ability value, emotional intelligence value or self-efficacy value, as shown in Formula 2-2. In the formula, *M* is the total number of hidden units, *p* = 1, 2…, *N* (*N* is the total number of samples in the cognitive and emotional feature sample set), and *w*_*i*_ is the connection weighted value between the *i-th* basis function and the output node (Chen et al., 1991). Finally, the calculated cognitive and emotional values are imported into the production expert system (PES) for processing to identify employees’ cognitive and emotional problems and provide corresponding solutions.

#### Production Expert System

The Production Expert System is an intelligent computer program that contains expert-level knowledge and experience in a specific field, which can simulate the reasoning and decision-making process of human experts to solve a range of sophisticated problems. This system expresses expertise based on rules, conditions, and actions, which includes a knowledge base, fact database and inference machine. It can be used to deal with diagnostic and prescriptive problems (Haag and Cummings, 2009). The Production Expert System is usually used for problem identification and analysis. The system first calls on the expert knowledge stored in the knowledge base. Then, the inference machine is used to compare and evaluate the cognitive and emotional values of employees participating in budgeting. Finally, the psychological states and problems of employees can be diagnosed. Under the premise of clarifying the characteristics of the employees’ psychological states, the system answers the mandatory questions, that is, analyzing, judging and reasoning about existing problems, drawing conclusions and storing them in the fact database. The system derives solutions through analysis and uses an inference machine to explain the analysis process and deliver final conclusions. It can provide managers with professional solutions and help them effectively guide employees’ cognitive and emotional activities.

#### Management Application Innovation System

As an innovative system based on human–machine coordination, the management application innovation system is jointly affected by human intelligence and artificial intelligence. It eliminates the traditional method of relying on managers’ personal knowledge and experience to make program decisions and creatively introduces artificial intelligence into management decision-making. By combining the solutions provided by PES with the manager’s own wisdom, we can design more valuable plans to solve employees’ cognitive and emotional problems. Furthermore, we are able to manage and control employees’ behaviors by guiding their cognitive and emotional activities. From the perspective of intelligent management, this system realizes man-machine interaction and has the compound effect of 1+1 > 2. The sum of man-machine intelligence produced by it will synchronously promote human intelligence and artificial intelligence.

The AI-based cognition and emotion identification system is an identification system composed of a number of independent subsystems that are interconnected and restricted to each other, forming a complex non-linear system. The system has learnability, flexibility and subjective initiative. These characteristics give the system strong adaptability in changing environments. The questionnaire data processing system mainly sets up items to objectively and accurately evaluate problems, classifies and sorts the data, and later transmits the effective data to the input layer of the neural network analysis system. As the core component of the non-linear system, the neural network analysis system is a three-layer feedforward neural network composed of an input layer, a hidden layer and an output layer. It can effectively simulate the human cognitive and emotional process and output the employees’ cognition and emotion values to the production expert system. The production expert system can simulate the thinking and reasoning processes of the brain, thereby clarifying the cognitive and emotional states of employees. It can assist managers in effectively intervening in employees’ cognitive and emotional activities. By analyzing and controlling their possible cognitive and emotional problems, corporate performance could be improved.

## Research Conclusion and Prospects

From the psychological and behavioral perspectives, this paper empirically examines the role of cognition and emotion in the relationship between participative budgeting and corporate performance. It is found that cognition and emotion can exert a positive effect from participative budgeting on corporate performance. On this basis, the AI-based cognition and emotion identification system is designed according to the theory of artificial intelligence. Through the cognition and emotion identification system, managers can identify the cognitive and emotional characteristics of budget participants and take necessary measures to intervene to improve corporate performance.

With the development of artificial intelligence, it is possible for people to evaluate cognitive and emotional characteristics through artificial intelligence systems and intervene with human psychology through management. With the continuous development of AI, its application in psychology and management is sure to expand. Additionally, the theoretical construction and practice of psychology and management will evolve.

## Data Availability Statement

The raw data supporting the conclusions of this article will be made available by the authors, without undue reservation.

## Ethics Statement

Ethical review and approval was not required for the study on human participants in accordance with the local legislation and institutional requirements. The patients/participants provided their written informed consent to participate in this study.

## Author Contributions

YZ: designing and writing. LL: designing. TZ, LZ, and ZX: writing. MB: processing data. All authors contributed to the article and approved the submitted version.

## Conflict of Interest

The authors declare that the research was conducted in the absence of any commercial or financial relationships that could be construed as a potential conflict of interest.

## Publisher’s Note

All claims expressed in this article are solely those of the authors and do not necessarily represent those of their affiliated organizations, or those of the publisher, the editors and the reviewers. Any product that may be evaluated in this article, or claim that may be made by its manufacturer, is not guaranteed or endorsed by the publisher.
